# The Impact of the Antimicrobial Compounds Produced by Lactic Acid Bacteria on the Growth Performance of *Mycobacterium avium* subsp. *paratuberculosis*

**DOI:** 10.3389/fmicb.2018.00638

**Published:** 2018-04-03

**Authors:** Petr Kralik, Vladimir Babak, Radka Dziedzinska

**Affiliations:** Veterinary Research Institute, Brno, Czechia

**Keywords:** bacteriocin, cell-free supernatant, paratuberculosis, pH, lactic acid, viability, propidium monoazide, real-time PCR

## Abstract

Cell-free supernatants (CFSs) extracted from various lactic acid bacteria (LAB) cultures were applied to *Mycobacterium avium* subsp. *paratuberculosis* (MAP) cells to determine their effect on MAP viability. In addition, 5% lactic acid (LA; pH 3) and commercially synthetized nisin bacteriocin were also tested. This procedure was chosen in order to mimic the influence of LAB compounds during the production and storage of fermented milk products, which can be contaminated by MAP. Its presence in milk and milk products is of public concern due to the possible ingestion of MAP by consumers and the discussed role of MAP in Crohn’s disease. Propidium monoazide real-time PCR (PMA qPCR) was used for viability determination. Although all CFS showed significant effects on MAP viability, two distinct groups of CFS – effective and less effective – could be distinguished. The effective CFSs were extracted from various lactobacilli cultures, their pH values were mostly lower than 4.5, and their application resulted in >2 log_10_ reductions in MAP viability. The group of less effective CFS were filtered from *Lactococcus* and enterococci cultures, their pH values were higher than 4.5, and their effect on MAP viability was <2 log_10_. LA elicited a reduction in MAP viability that was similar to that of the group of less effective CFS. Almost no effect was found when using commercially synthetized nisin at concentrations of 0.1–1000 μg/ml. A combination of the influence of the type of bacteriocin, the length of its action, bacteriocin production strain, and pH are all probably required for a successful reduction in MAP viability. However, certain bacteriocins and their respective LAB strains (*Lactobacillus* sp.) appear to play a greater role in reducing the viability of MAP than pH.

## Introduction

A variety of Gram-positive and Gram-negative bacteria are capable of producing compounds with antimicrobial activity that inhibits the growth of other bacteria – bacteriocins. Simple bacteriocins are chemically small- or medium-sized peptides or proteins. They are ribosomally synthetized and usually undergo post-translational modification. Their activity is directed against related species or even against bacteria of the same strain (Zacharof and Lovitt, 2012). Bacteriocins are synthetized by many bacterial species, including lactic acid bacteria (LAB). Apart from bacteriocins, LAB produce organic acids, such as lactic and acetic acids, hydrogen peroxide, or diacetyl. All these compounds have antimicrobial properties, which underlies the use of LAB in the food industry as inhibitors of food-borne pathogens (probiotic cultures). LAB bacteriocins have the potential to be used in food biopreservation (Galvez et al., 2007). At this time, only nisin, as the most well-studied and best-known bacteriocin, has been approved for use in the food industry (O’Sullivan et al., 2002).

*Mycobacterium avium* subsp. *paratuberculosis* (MAP) causes an infectious and chronic disease in ruminants called paratuberculosis. The clinical phase is characterized by diarrhea, emaciation, and low milk yield, and is preceded by a long, latent phase (years) during which other, healthy animals can be infected. Therefore, the disease is mostly of importance in cattle herds. Infected animals can shed MAP in milk (Slana et al., 2008). This is of public concern due to the possible ingestion of MAP and its potential link to Crohn’s disease (Hermon-Taylor, 2009; Chiodini et al., 2012). In the past, many studies investigated the ability of MAP to survive unfavorable conditions in dairy products; however, their results were not always consistent. Ability of MAP to survive pasteurization process was demonstrated in many studies (Grant et al., 2002; Carvalho et al., 2012), but the others did not confirm it (Serraino et al., 2017). Similarly, it applies to the fermentation process. MAP is capable to surive fermentation (Donaghy et al., 2004; Hanifian, 2014), but on the other hand, it is evident that its growth is suppressed during the fermentation and storage of milk products such as cheese (Spahr and Schafroth, 2001; Donaghy et al., 2004; Hanifian, 2014) or yogurts (Van Brandt et al., 2011; Klanicova et al., 2012). As the role of MAP in Crohn’s disease is still not clear, reducing exposure of consumers thought reducing MAP contamination in food is desirable.

Utilization of propidium monoazide treatment followed by real-time PCR based on the amplification of the *F57* fragment (PMA qPCR) was previously employed to study MAP viability after exposure to diverse conditions (Pribylova et al., 2012; Kralik et al., 2014). The method combines the advantages of cultivation and qPCR. Results are obtained quickly, and the method is sensitive, specific, and provides quantitative data. The PMA dye selectively penetrates dead cells with compromised membranes and covalently binds to its DNA. After photoactivation of the dye (exposure to bright light), it forms insoluble complex with DNA and prevents its amplification in subsequent PCR. At the same time, the excess dye, which has not penetrated into the damaged cells, is inactivated by light. The portion of viable cells is then calculated as the quotient of PMA-treated stressed cells and the control (Nocker et al., 2006; Kralik et al., 2010). Previous reports have shown that the experimentally determined difference between completely live and dead (heat-treated) MAP cells using PMA qPCR is about 2 log_10_ (Kralik et al., 2010). As viability determination using culture and PMA qPCR has been described to yield comparable results, the latter can be applied for the determination of MAP viability as the sole method (Pribylova et al., 2012).

In previous publications, the effect of whole milk products (yogurts, acidophilus milk, etc.) containing LAB and the products of their metabolism on the viability of mycobacteria was studied (Van Brandt et al., 2011; Klanicova et al., 2012). The aim of this current study was to assess the viability of three MAP isolates after exposure to filtered cell-free supernatants (CFSs) acquired by the propagation of LAB cultures in their respective media in accordance with standard culture procedures. This procedure was chosen in order to mimic the influence of LAB compounds during the production and storage of fermented products. In addition to CFS, the effects of different concentrations of commercially synthetized nisin and one concentration of lactic acid (LA) on MAP viability were assessed. The viability of MAP strains and isolates was measured using the culture-independent method consisting of treatment with PMA combined with specific qPCR.

## Materials and Methods

### Bacterial Isolates and Culture Conditions

The MAP laboratory strain CAPM 6381 (The Collection of Animal Pathogenic Microorganisms, Veterinary Research Institute, Czechia) and field isolates 7082 (white deer) and NL-3 (cattle) were used throughout the whole study. All strains and isolates were of RFLP type C1.

All strains and isolates were grown on Herrold’s egg yolk medium (HEYM) with penicillin G, chloramphenicol, and amphotericin B (Becton Dickinson, Franklin Lakes, NJ, United States), supplemented with 2 μg/ml Mycobactin J (Allied Monitor, Fayette, MO, United States) at 37°C for 2–3 months until visible colonies were observed. Afterward, grown colonies were harvested using a loop and resuspended in 1.5 ml of Middlebrook 7H9 (M7H9) broth supplemented with 10% (vol/vol) Middlebrook OADC enrichment (both Becton Dickinson, Franklin Lakes, NJ, United States). In order to homogenize the MAP suspensions, 12 1-mm zirconia/silica beads (Biospec, Bartlesville, OK, United States) were added followed by vortexing for 30 s. To remove the MAP clumps, the suspension was centrifuged at 100 × *g* for 30 s and the upper cell fraction was resuspended in fresh M7H9 broth. The suspension was diluted to OD_600_ ≈ 0.15–0.20, which corresponds to approximately 10^8^ MAP cells/ml of suspension (Kralik et al., 2011).

### Preparation of Milk Starter Cultures for CFS

Nine bacterial strains used as milk starter cultures originating from the Lactoflora^®^ collection of milk cultures (Milcom, Prague, Czechia) were propagated in their respective media – M17 and MRS broth (both Oxoid CZ, Brno, Czechia). Each culture was characterized in detail with respect to its culture conditions, active acidity, and production of bacteriocins (**Table 1**). The type of bacteriocin was determined by the well method, PCR, or a combination of both. Each milk starter culture was cultured in 250 ml of its respective medium for 18 h. Afterward, the culture of starter bacteria was filtered through a 0.22-μm microbiological filter to prepare CFS. The sterilized, filtered CFSs were subsequently applied to MAP strains and isolates to determine their influence on MAP viability over time.

**Table 1:** Characteristics of milk starter cultures used in this study.

No.	Strain	Species	Culture conditions		Active acidity	Bacteriocin production
S1	CCDM 731	*Lactococcus lactis* subsp. *Lactis*	37°C 18 h	M17 broth	4.65	Nisin
S2	CCDM 857	*Enterococcus faecalis*		M17 broth	4.51	Enterocin
S3	CCDM 108	*Lactobacillus helveticus*		MRS broth	4.14	Enterocin
S4	RL 26-P	*Lactobacillus plantarum*		MRS broth	3.67	Plantaricin
S5	CCM 7165	*Enterococcus faecium*		M17 broth	4.68	Enterocin
S6	RL 23-P	*Lactobacillus* sp.		MRS broth	4.11	Plantaricin
S7	CCDM 768	*Lactobacillus helveticus*		MRS broth	4.07	Enterocin
S8	CCDM 182	*Lactobacillus plantarum*		MRS broth	3.81	Plantaricin
S9	RL 22-P	*Lactobacillus gasseri*		MRS broth	4.51	Plantaricin


### Nisin and LA Preparation

In order to mimic the effect of bacteriocins and reduced pH separately, MAP suspensions were exposed to nisin and solutions of LA. Commercially available nisin (Sigma, St. Louis, MO, United States) was diluted in water and added to the M7H9 media to prepare solutions with final concentrations of 0.1, 1, 10, 100, and 1000 μg of nisin/ml of M7H9. A solution of LA was diluted in M7H9 media to a concentration of 5% (v/v) and the pH was adjusted to 3. This value was chosen with respect to the pH of CFS and previous studies (Klanicova et al., 2012).

### Exposure of Antimicrobial Substances on the Viability of MAP Strains and Isolates

On day 0, 6 ml of each of the three MAP strains and isolates prepared as described above was aliquoted into 16 tubes (nine milk culture starters, five concentrations of nisin, LA treatment, and control) in biological triplicates. All the tubes were centrifuged at 7000 × *g* for 3 min and the supernatant was replaced by 6 ml of filtered CFS, solutions of nisin, and LA (or respective medium – MRS or M17 broth as a control sample). All the samples were placed in an incubator set at 37°C. In order to mimic the influence of antimicrobial compounds on MAP viability during the storage of the fermented product, all samples were taken on days 4, 8, and 12.

### PMA Treatment

The viability of MAP after exposure to conditions suppressing bacterial growth, which are induced by fermentation and last during the production and maturation of the fermented product, was determined by the PMA procedure combined with subsequent *F57* qPCR (Kralik et al., 2010, 2014; Pribylova et al., 2012). On each sampling day (days 4, 8, and 12), 3× 0.5-ml aliquots were taken from each sample (technical triplicates). All samples were centrifuged (7000 × *g* for 3 min) and the supernatant was replaced with fresh medium. To each 500 μl sample (including controls), 12.5 μl of 1 mM PMA (Biotium Inc., Hayward, CA, United States) stock solution dissolved in 20% DMSO was added (final concentration 25 μM). The mixture was incubated in the dark for 5 min with mixing at 20 Hz and then placed on ice and exposed to light from a 650-W halogen bulb (B&H Photo Video, New York, NY, United States) for 2 min. The whole process of incubation in the dark and light exposure was repeated once again with a freshly added aliquot of PMA. After the PMA treatment, the samples were centrifuged at 7000 × *g* for 3 min and the supernatant was replaced with 500 μl of Tris-EDTA (TE) buffer supplemented with 50 ng/μl of fish sperm DNA (both SERVA Electrophoresis, Heidelberg, Germany). The MAP cells were lysed by incubation at 100°C for 20 min, and after centrifugation at 18,000 × *g* for 5 min, the supernatant served as a template for the qPCR.

### F57 qPCR

Quantification of MAP cells for the purposes of viability determination was performed using a qPCR assay amplifying the single copy fragment *F57* according to a calibration curve based on serial dilution of a plasmid gradient on a LightCycler 480 instrument (Roche Molecular Diagnostic, Germany; Slana et al., 2008). MAP viability was determined as the quotient of absolute numbers of PMA-exposed stress factor-treated cells (treated with CFS, nisin, or LA) and the PMA-exposed control (stress factor untreated; MAP in the respective broth) cells (Kralik et al., 2010, 2014; Pribylova et al., 2012).

### Statistical Analysis

All the statistical calculations were performed using Statistica 13.0 software (StatSoft, Tulsa, OK, United States). Three-way ANOVA (with factors CFS, time, strain and CFS, time, concentration, respectively) was used for statistical evaluation of the effect of CFS and nisin, respectively, on MAP strain viability. For the determination of LA influence on MAP viability, two-way ANOVA was used (with factors time and strain). *P*-values <0.05 were considered statistically significant.

## Results

### Effect of CFS on MAP Viability

**FIGURE 1:**
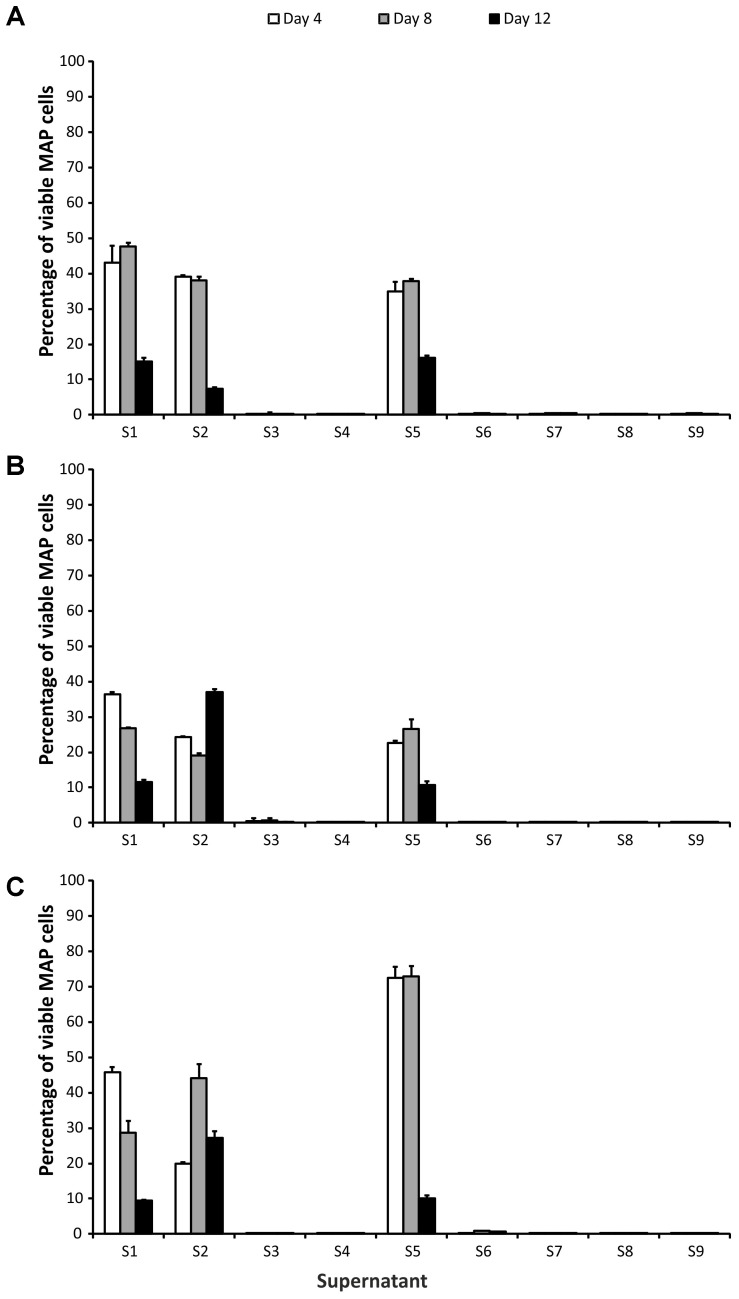
Effect of supernatants on *Mycobacterium avium* subsp. *paratuberculosis* (MAP) viability. **(A)** Field isolate 7082 (white deer); **(B)** collection strain 6381; **(C)** field isolate NL-3 (cattle). Vertical bars represent sample standard deviations. The portion of viable cells is calculated as the quotient of PMA-treated stressed cells (supernatants) and the PMA-treated control (stress factor untreated; MAP in the respective broth).

Based on their impact on MAP viability, two groups of CFS could be distinguished. Application of S3, S4, S6, S7, S8, and S9 CFS caused a significant reduction in the number of surviving cells (at least 2 log_10_, i.e., below 1%), while in the case of the group containing S1, S2, and S5 CFS the viability reduction was about maximum 1 log_10_ (**Figure 1**). CFS could therefore be divided into effective (S3, S4, S6, S7, S8, and S9) and less effective (S1, S2, and S5), with a discriminatory value for classification into one or the other group of 1% (this corresponds to an average decrease in the number of surviving cells of 2 log_10_). To demonstrate the effect of effective and less effective CFS, ANOVA with three factors (CFS, time, and strain) was performed.

In the group of effective CFS, it was shown that while there was no statistically significant difference between the strains (*P* > 0.05, *F*-test), the CFS and the time factor were statistically significant variables (*P* < 0.01 and *P* < 0.05, respectively). The interaction of CFS and strain was also significant (*P* < 0.05). Subsequent testing confirmed that there was a significant difference between S4 and S6 (*P* < 0.05; Tukey’s HSD test) and between S6 and S8 (*P* < 0.05). In the case of the time factor, a statistically significant difference between days 4 and 8 (*P* < 0.05) was demonstrated.

No difference was found among the CFS in the less effective group (*P* > 0.05; *F*-test). The strain and the time factor were statistically significant variables here (both *P* < 0.01) as well as all factor interactions. Significant differences between NL-3 and 6381 (*P* < 0.01; Tukey’s HSD test) and 6381 and 7082 (*P* < 0.05) isolates were proven (**Figure 1**). Statistically significant differences between days 4 and 12 and between days 8 and 12 (in both cases *P* < 0.01) were also evident (**Figure 1**).

### Effects of Nisin and LA on MAP Viability

**FIGURE 2:**
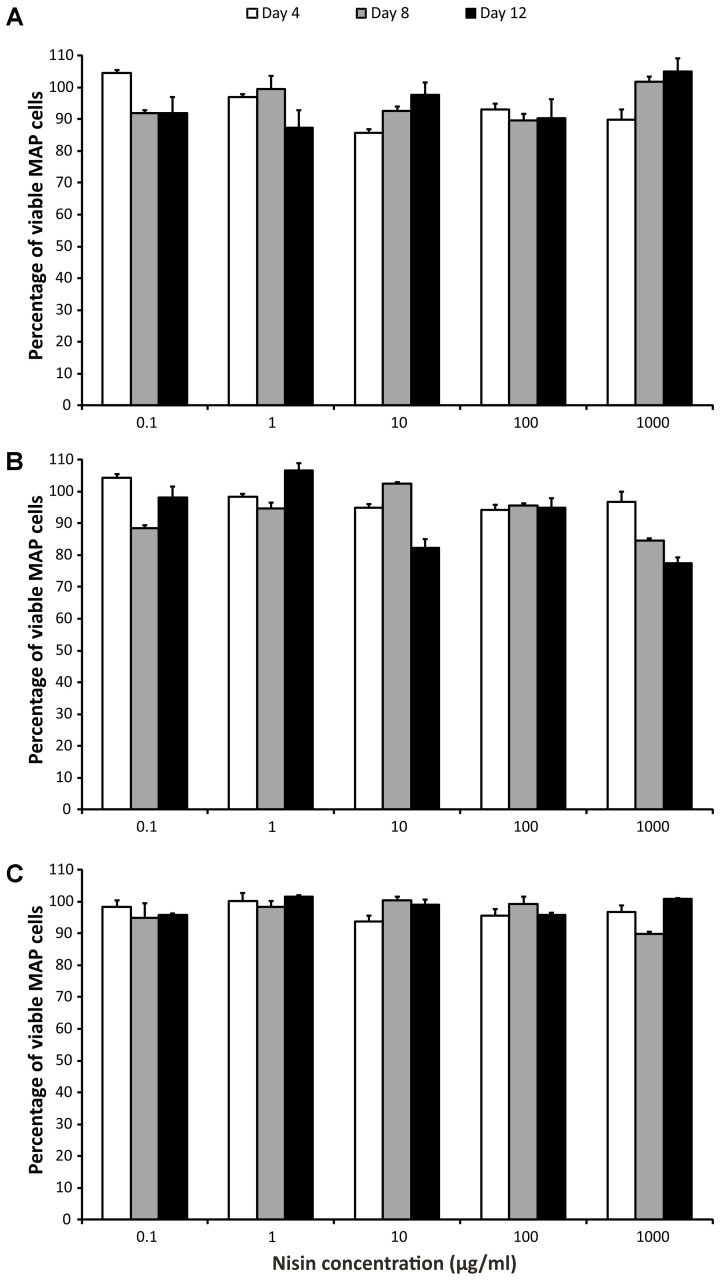
Effect of nisin on MAP viability. **(A)** Field isolate 7082 (white deer); **(B)** collection strain 6381; **(C)** field isolate NL-3 (cattle). Vertical bars represent sample standard deviations. The portion of viable cells is calculated as the quotient of PMA-treated stressed cells (nisin) and the PMA-treated control (stress factor untreated; MAP in the respective broth).

Three-way ANOVA (time, strain, and concentration) was used to estimate the influence of nisin on MAP viability. There was no statistically significant impact of nisin exposure on any of the MAP isolates, despite the fact that the concentrations of nisin covered a larger than 5 log_10_ range (10^-1^–10^3^ μg/ml; **Figure 2**).

**FIGURE 3:**
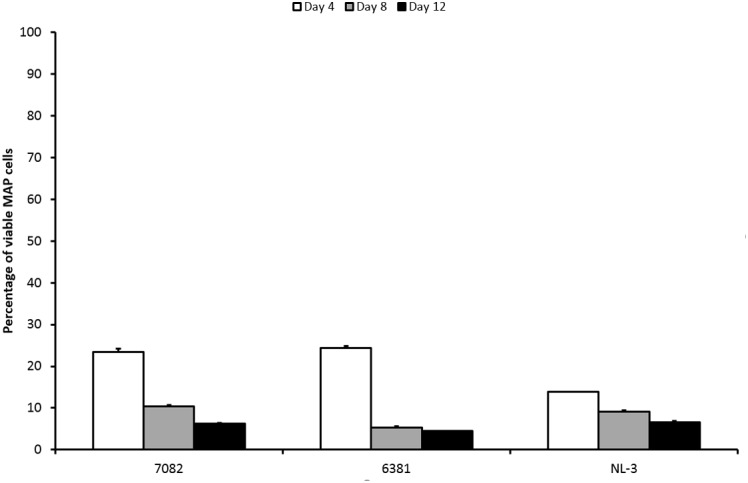
Effect of LA on viability of three MAP isolates. Vertical bars represent sample standard deviations. The portion of viable cells is calculated as the quotient of PMA-treated stressed cells (LA) and the PMA-treated control (stress factor untreated; MAP in the respective broth).

The percentage of surviving MAP cells was reduced by less than 2 log_10_ after LA exposure. With respect to CFS, the LA would thus be described as less effective. The percentage of surviving cells decreased over time for all strains (**Figure 3**). Both strain and time factors were statistically significant (*P* < 0.01; *F*-test). Difference between days 4 and 8 and between days 4 and 12 was confirmed (*P* < 0.01; Tukey’s HSD test). The response of the NL-3 isolate was significantly different to those of CAPM 6381 and 7082 isolate (*P* < 0.01 for NL-3 and 7082). LA was approximately twice as effective in killing NL-3 MAP cells after 4 days compared with the other two isolates. At later stages of the experiment (days 8 and 12), the efficacy of MAP cell killing was equal in all strains (**Figure 3**).

## Discussion

All CFS exerted a significant effect on MAP viability. However, their effects were either more (S3, S4, S6, S7, S8, and S9) or less effective (S1, S2, and S5). No viable MAP were found after 4 days of culture with effective CFS. The effective CFSs were products of *Lactobacillus* sp., while enterococci and *Lactococcus lactis* subsp. *lactis* were producers of the less effective CFS group (**Table 1**). In the previous study, addition of CFS from a number of *Lb. paracasei* isolates delayed the growth of MAP. Application of CFS from *Lb. casei* and *Lb. rhamnosus* led to suppression of the increase in growth typical for MAP metabolic activity (Donaghy et al., 2005). Glass et al. (2004) showed CFS from *Lb. acidophilus* and *Lb. reuteri* significantly decreased viability of vegetative cells and spores of *Cryptosporidium parvum*. Gaggia et al. (2010) also tested the effect of various CFS derived from LAB on MAP viability; one strain of *Lb. plantarum* elicited a significant decrease in MAP intracellular pH and led to loss of its viability. The *Lb. plantarum* strain produced plantaricin, while the others, not effective LAB strains contained enterocins (Gaggia et al., 2010). In consistence with Gaggia et al. (2010), plantaricins were exclusively covered in the group of effective CFS in our case and showed significant decrease in MAP viability. On the other hand, the group of effective CFS also included enterocins, which were nevertheless covered in the group of less effective CFS as well (**Table 1**). It is known that different enterocins show different inhibitory spectra toward selected LAB and pathogens (Moreno et al., 2003). This could explain different effect of various enterocins on MAP viability within this study (effective and less effective CFS). The differing inhibitory effects of enterocins on MAP could probably be also explained by different LAB producers. While effective enterocins (and plantaricins as well) were produced only by lactobacilli (*Lb. helveticus*, *Lb. plantarum*, *L. gasseri*, and *Lb.* sp.), less effective enterocins and nisin were synthetized by *Lactococcus* and enterococci (**Table 1** and **Figure 1**).

pH values differed slightly between the two groups. Values of around 4 or slightly lower were observed in the effective group of CFS (except for S9 with its limit value), while values between 4.5 and 5.0 were measured in the less effective group. The correlation between pH and bacteriocin activity was observed previously by Moreno et al. (2003). They found that the activity and stability of enterocins are higher at low pH. This can be ascribed to the solubility of enterocins which is increasing with decreasing pH (Moreno et al., 2003). Klanicova et al. (2012) described a significant inhibition of MAP growth when pH values of fermented milk products dropped below 4. The production of organic acids and subsequent lowering of pH were shown to be the main inhibitory mechanism of bifidobacteria toward Gram-negative bacteria in another study. According to the authors, the bacteriocins of bifidobacteria have only negligible effect on Gram-negative bacteria compared to the production of organic acids and lowering of pH (Makras and De Vuyst, 2006). On the contrary, another study suggested that the inhibitory effect of lactobacilli themselves rather than acid production is responsible for MAP growth delay (Donaghy et al., 2005). The same observations were made by Van Brandt et al. (2011) who did not note any changes in MAP counts after storage of commercial yogurts (low pH) with inoculated MAP. Thus, the question of whether bacteriocins or pH have the critical impact on MAP viability is still open. Based on the results with CFS themselves, it seems that the type of bacteriocin, the length of its action, LAB production strain, and pH are all involved in the inhibition of MAP growth.

A vast range of nisin concentrations (0.1–1000 μg of nisin/ml media) was selected to cover the maximal range of concentrations. Nevertheless, there was almost no decrease in MAP viability at any concentration (**Figure 2**). In the study using alamarBlue reagent, the effect of various concentrations of nisin on *M. kansasii*, *M. tuberculosis*, and MAP viability was examined. Nisin showed good efficacy against MAP, but 60 μg/ml, the highest concentration tested, was below the MIC_90_ for MAP and *M. tuberculosis* (Carroll et al., 2010). Inhibition of *M. smegmatis* growth was observed at a nisin concentration of 10 μg/ml; a much higher concentration (2500 μg/ml) was needed for *M. tuberculosis* (Chung et al., 2000). By measuring intracellular pH, a large decrease in viable MAP cells using 2.5 kU/ml (recounted as 2500 μg/ml) of nisin was showed. After 30 min, no viable cells were detected (Gaggia et al., 2010). According to these previous findings, it seems that nisin concentrations markedly above 1000 μg/ml would be needed for any substantial decrease in MAP viability. However, taking into account the study of Carroll et al. (2010), at least some reduction in MAP viability had to be attained, but this was not demonstrated at all (**Figure 2**). Thus, another interpretation can be used to explain the almost 100% maintenance of viability after several days of treatment with even high concentrations of nisin. The PMA qPCR method is based on the penetration of PMA through pores in bacterial membranes caused by the action of antimicrobial agents. It is known that nisin binds to cytoplasmic membranes and forms pores by inserting through membranes. It was found that pores formed by nisin in *M. bovis* BCG were large enough to leak protons, but too small for ATP (Chung et al., 2000). With respect to the relatively large size of the PMA molecule and the small pores formed by nisin, a possible inability of PMA to penetrate through these pores should be considered.

The mechanism of LA antimicrobial activity is based on the passing of its undissociated forms through the cell membrane. Increased cytoplasmic pH leads to the dissociation of the acid, the release of protons, and subsequent acidification of the cytoplasm (Cotter and Hill, 2003; Reis et al., 2012). It is known that mycobacteria have higher tolerance to acidic environments compared to other bacteria (Cotter and Hill, 2003). MAP cells remained culturable for at least 7 days at pH 4 (Cook et al., 2013). In fermented milk products with pH values of around 4, MAP remained culturable for even up to 6 weeks (Klanicova et al., 2012). In our study, 5% LA with pH 3 played a role in the reduction of MAP viability; however, it had only a moderate effect. This was evidenced by the presence of 14–25% viable MAP cells after 4 days and at least 5% of viable MAP after 12 days of LA treatment (**Figure 3**). With respect to CFS, the effect of LA was comparable to the group of less effective CFS. However, the pH of the LA (pH 3) was closer to the pH value of the effective CFS (below or around 4 mostly) than to the less effective ones (above 4.5). If the pH should have a decisive impact on reducing MAP viability, then its effect should be rather comparable to the group of effective CFS. Thus, it can be assumed that although pH plays an important role in reducing the viability of MAP, a significant decrease in viability below 1% is due to bacteriocins or LAB themselves than to pH.

The effect of LA on 6381 and 7082 strains was not as considerable as on NL-3 after the first 4 days of treatment (**Figure 3**). While NL-3 is a field isolate, 6381 is a laboratory strain. MAP 7082 has gone through many passages in the laboratory and therefore it can be expected that its response will rather correspond to the response of laboratory strain. In general, laboratory strains show higher tolerance to stress factors comparing to field isolates (Gumber and Whittington, 2007; Kralik et al., 2014), which could explain more successful decrease of viability in NL-3 compared to 6381 and 7082 (**Figure 3**). Totally different situation, however, occurred with the NL-3 after its exposure to the CFS No. 5. After 4 and 8 days of the treatment, CFS No. 5 showed negligible effect on NL-3 compared to other CFS from the less effective group (Nos. 1 and 2; **Figure 1**). Unexpectedly, NL-3 also showed significantly higher tolerance to CFS No. 5 compared to laboratory strain 6381 and high-passage strain 7082 (**Figure 1**). It is unclear why only the effect of CFS No. 5 on the NL-3 viability was so insignificant, and further investigation would be required to clarify this.

To sum up, the effect of filtered CFS acquired by the propagation of LAB cultures, commercially synthetized nisin, and one concentration of LA to the viability of MAP isolates was investigated. All nine CFS showed significant effects on MAP viability. Nevertheless, two distinct groups of effective and less effective CFS could be distinguished. The effective CFSs comprising of six bacteriocins (plantaricins and enterocins) were extracted from various lactobacilli cultures (*Lb. helveticus*, *Lb. plantarum*, *L. gasseri*, and *Lb*. sp.) and their pH values were mostly around 4 (3.6–4.5). Application of these CFS resulted in >2 log_10_ reduction in MAP viability (below 1% of surviving) and no viable MAP were observed after 4 days of treatment. The group of less effective CFS (enterocins and nisin) were filtered from *L. lactis* subsp. *lactis* and enterococci cultures (*E. faecalis* and *E. faecium*) and their pH values were higher than 4.5. Less effective CFS elicited a reduction in MAP viability of <2 log_10_. LA applied at a concentration of 5% (pH 3) resulted in a reduction in MAP viability similar to that of the less effective CFS group. After 4 days of treatment, about 14–25% of MAP cells survived; 5% of viable cells was still found after 12 days of LA treatment. On the contrary, almost no effect was found when using commercially synthetized nisin at concentrations of 0.1–1000 μg/ml. It seems that the type of bacteriocin, the length of its action, LAB production strain, and pH are probably all involved in determining the success of MAP viability reduction. Nevertheless, as the effect of LA was comparable to the group of less effective CFS than those effective, it can be assumed that certain bacteriocins and their LAB production strains could play more important role in reducing the viability of MAP than the pH.

## Author Contributions

PK was in charge of the whole project and participated in data production, data interpretation, and drafting the manuscript. VB designed and performed the statistical analysis and revised the paper critically. RD interpreted the data and wrote up the paper. All authors read and approved the final manuscript.

## Conflict of Interest Statement

The authors declare that the research was conducted in the absence of any commercial or financial relationships that could be construed as a potential conflict of interest.
